# Layer-by-Layer Coating of Single-Cell *Lacticaseibacillus rhamnosus* to Increase Viability Under Simulated Gastrointestinal Conditions and Use in Film Formation

**DOI:** 10.3389/fmicb.2022.838416

**Published:** 2022-05-04

**Authors:** Maram Sbehat, Mohammad Altamimi, Mohammad Sabbah, Gianluigi Mauriello

**Affiliations:** ^1^Department of Nutrition and Food Technology, An-Najah National University, Nablus, Palestine; ^2^Department of Agricultural Sciences, University of Naples Federico II, Naples, Italy

**Keywords:** layer-by-layer, coating, probiotics, *Lactobacillus rhamnosus*, food waste protein

## Abstract

Probiotics and prebiotics are widely used as functional food ingredients. Viability of probiotics in the food matrix and further in the digestive system is still a challenge for the food industry. Different approaches were used to enhance the viability of probiotics including microencapsulation and layer-by-layer cell coating. The of aim of this study was to evaluate the viability of coated *Lacticaseibacillus rhamnosus* using a layer-by-layer (LbL) technique with black seed protein (BSP) extracted from *Nigella sativa* defatted seeds cakes (*Ns*DSC), as a coating material, with alginate, inulin, or glucomannan, separately, and the final number of coating layers was 3. The viable cell counts of the plain and coated *L. rhamnosus* were determined under sequential simulated gastric fluid (SGF) for 120 min and simulated intestinal fluid (SIF) for 180 min. Additionally, the viability after exposure to 37, 45, and 55°C for 30 min was also determined. Generally, the survivability of coated *L. rhamnosus* showed significant (*p* ≤ 0.05) improvement (<4, 3, and 1.5 logs reduction for glucomannan, alginate and inulin, respectively) compared with plain cells (∼6.7 log reduction) under sequential exposure to SGF and SIF. Moreover, the cells coated with BSP and inulin showed the best protection for *L. rhamnosus* under high temperatures. Edible films prepared with pectin with LbL-coated cells showed significantly higher values in their tensile strength (TS) of 50% and elongation at the break (EB) of 32.5% than pectin without LbL-coated cells. The LbL technique showed a significant protection of probiotic cells and potential use in food application.

## Introduction

The World Health Organization (WHO) has defined probiotics as “live microorganisms which when administered in adequate amounts confer a health benefit on the host” (FAO/WHO, 2001). Later, the definition was reworded as “live microorganisms that, when administered in adequate amounts, confer a health benefit on the host” (Hill et al., 2014).

The term probiotics includes strains belonging to several genera of bacteria and yeasts, such as *Lactobacillus*, *Bifidobacterium*, *Streptococcus*, *Enterococcus*, *Escherichia*, *Bacillus*, and *Saccharomyces*. These microorganisms could be naturally found in fermented food like yogurt and dairy products, pickles, sauerkraut, kefir, Kombucha, etc. (Dimidi et al., 2019). Besides, they are available in the market in the form of probiotic foods or as supplements. *Lacticaseibacillu rhamnosus* originally isolated from humans has attracted more attention due to its ability to survive stomach acids and bile salts (Segers and Lebeer, 2014), and to its positive effect on the progression of diseases including gastrointestinal (GI) infections (Capurso, 2019).

The interest of probiotics and its introduction to food is massively increased during the past few years owing to reported evidence about probiotics’ positive effect on the human health. Probiotics promote health status and play an essential role against colonization of pathogenic microbes in the intestine by the production of antimicrobial compounds, increase gut integrity by stimulating mucus production, improve enzyme formation, regulate the composition of (GI) microbiota, and act as immunity modulators. Nowadays, the investigations about the importance of GI microbiota are widely and intensively increased. It was reported that probiotics and GI microbiota composition demonstrate a major role in the progression of many diseases and disorders, such as obesity, allergies, diabetes, inflammations, inflammatory bowel diseases (IBD), cancer, infectious diseases, and even neurodegenerative diseases (Kechagia et al., 2013).

Therefore, the food industry has never missed the chance to increase its profits by producing novel probiotics. From the economic point of view, the global probiotics market is expected to reach USD 77.09 billion by 2025 (Grand View Research, 2021). Moreover, economic analysts have expected an increase in the demand for functional food especially during the COVID-19 pandemic. It has been demonstrated that certain probiotics, such as lactic acid bacteria, produce bioactive peptides, such as angiotensin-converting enzyme inhibitor, which may help manage COVID-19 (Meng et al., 2020; Bhattacharya et al., 2021).

This increase is owing to consumer’s awareness about the benefits of probiotics and its effect on health and, particularly, immunity boosting, and their tendency toward healthier food in general. On the other hand, analysts have expected a depression in the consumption of meat, poultry, and seafood, while the demand for plant and animal protein supplements will increase (Grand View Research, 2021). This may affect the distribution of food items that used to be probioticated, or even under ongoing researches. Furthermore, consumers perceive probioticated food as natural rather than taking it in pharmaceutical forms, i.e., pills, capsules, etc. (Fenster et al., 2019).

To achieve better usefulness, we need to be sure that the probioticated products have taken into account the differences in needs among all population categories, and the consumption of that product in a specific market. For example, (1) if we probioticate bread, we should consider the celiac disease population and gluten-free dieters, (2) milk product probiotication should take into account lactose-intolerant patients, so we think about the probiotication of alternatives suitable for this category, such as almond or soy milk, and (3) take in consideration the coating agents in case of vegan consumers (Grand View Research, 2021). Nevertheless, choosing the right probiotics for specific health conditions is still a challenge for some reasons, such as variations in the mode of action of different probiotics, differences in manufacturing processes, quality control of the products, and differences in the international regulatory requirements of minimum effective dose (Sniffen et al., 2018).

To obtain the maximum health benefits claimed about probiotics, the number of viable probiotic cells should be not lower than 10^6^–10^7^ cfu/ml or g ingested according to the Food and Drug Administration—WHO (Thomas et al., 2014). Unfortunately, the numbers of probiotics ingested are affected by numerous factors, including (1) chemical: Low pH, gastrointestinal conditions, food matrix properties, and (2) physiological factors that affect the adhesion and colonization of probiotics in the intestines, such as rapid transit time. Moreover, probiotics viability may be decreased under thermal conditions during their preparation or shelf life. Therefore, formulations of probiotics should consider the various physicochemical, biopharmaceutical, and biological barriers to make the best of their therapeutic effectiveness and clinical applicability (Baral et al., 2021).

To overcome these problems, the scientists thought of several strategies to improve probiotics viability, including nanoparticles, polymer gels, and microencapsulation to protect the sensitive probiotic cells against the harsh conditions in the gastrointestinal tract and during storage, and to improve the adhesion to the mucosal lining.

In addition, scientists have developed techniques to perform microencapsulation and obtain a proper form of final product with higher viability and stability. These techniques, including extrusion, emulsion, spray drying, spray freezing, spray-freeze drying, and layer-by-layer were investigated (Călinoiu et al., 2019).

The inclusion of prebiotics in such systems played several crucial roles, as these prebiotics increase the stability of the applied systems, help in developing of new food products, and further protect probiotics from harsh conditions. Moreover, prebiotics microencapsulation-based formulations have enhanced the nutritional and functional properties of the final products (Shah et al., 2020).

Layer-by-layer, as a technique, was raised up a few decades ago and proposed by the German chemist Gero Decher in 1991 (Matsusaki et al., 2012). At first, the applications of this technique have been limited in increasing the effectiveness of drugs and pharmaceuticals; then it was outspread until it linked to food processing by coating some types of food pigments and single-cell probiotics (Ram et al., 2001; Priya et al., 2011; Marais et al., 2014; Jung et al., 2015).

The layer-by-layer technique is described by electrostatic attractions and ionic interactions between the layers used in the coating. This approach is based mainly on the alternate exposure of the probiotic cell to negative- and positive-charged substrates. In order to remove the excess of the coating materials, a buffer solution is used as a washing solution after each coating step. In the end, the coated probiotics might be dried depending on the application (Priya et al., 2011).

The main negative-charged molecules used in the coating of probiotics were alginate and pectin polysaccharides, while the studies on the positive-charged layer were covering (1) polysaccharides as chitosan and (2) proteins as milk proteins or, as in the current study, protein concentrate extracted from *Nigella sativa* defatted cake (*Ns*DSC), which is widely available as a byproduct after oil extraction. Moreover, the compounds used in LbL must be of food grade, for example, biodegradable polysaccharides, and able to tolerate harsh conditions, such as acidic pH, bile salts, digestive enzymes, and storage conditions, including temperature (Alkekhia et al., 2020; Ivanova et al., 2020).

This technique steps ahead of microencapsulation techniques, fulfills the maximum saving of coating materials, and minimizes the wasting that occurs in the bead-form microcapsule. It also allows delivering of probiotics cells, growth, and colonization in the GIT with no need to release apart from the coating material.

Generally, all studies previously published approved the positive effect of LbL on the protection of probiotics in bacteria and yeast. Particularly, the LbL approach also enhanced the viability of probiotics under harsh conditions as detailed in Table 1.

**TABLE 1:** Effect of layer-by-layer of probiotics on its viability under harsh conditions.

References	+ve layer	-ve layer	Probiotic	Results		
				Gastrointestinal	Adhesion	Heat treatment
Xiao et al. (2020)	Whey protein isolates (WPI)	Xanthan Gum	*L. bulgaricus* *L. paracasei*	-Mortality of the cells decreased sig. -Survivability further increased when the micro-capsules coated with xanthan gum. -Coated *L. paracasei* showed higher resistance than L. bulgaricus		-Free cells: viable count decreased 1, 4 and 6 log cycle at 55, 65, and 75°C for 10 min, respectively. -Coated: less than 0.5, 1, and 3 log cfu/ml reduction at same temperatures, respectively.
Wang et al. (2019)	Chitosan	Sodium phytate	*L. pentosus*	-Plain: 7.4 and 6.09 log cfu/ml reduction in SGF for 2 h, and bile salts for 3 h, respectively. - Coated: 4.34 and 2.33 log cfu/ml reduction under the same conditions, respectively.		At 45°C, 1 and 0.5 log cfu/ml reduction were provided for plain and coated, respectively. At 65°C, no growth of plain cells while 3.2 log cfu/ml of coated cells were viable.
Anselmo et al. (2016)	Chitosan	Alginate	*Bacillus coagulans*	(CHI/ALG)1 showed protection against both bile salts and gastric fluids (4 log reduction) (CHI/ALG)2 → 1 log reduction	(CHI/ALG)2 BC exhibited nearly 1.5-fold higher adherence to mucosal surface of porcine intestines compared to plain-BC	–
Thomas et al. (2014)	Chitosan	Dextran sulfate	*S. boulardii*	-Almost 2 log and 0.5 log cycles reduction after 2 h SGF for plain and coated LP, respectively. -Coated cells showed a reduction in viability under bile salts but not as drastic as uncoated cells.		
Priya et al. (2011)	Chitosan	Carboxymethyl cellulose (CMC)	*L. acidophilus*	Survival of 1 and 6.3 log cfu/500 mg of plain and coated LA, respectively, after sequential exposure to 2 h of SGF, 2 h bile salts.		79 and 93% survival of plain and coated cells, respectively, after freeze drying.

The aim of this study was to coat probiotic single-cell *L. rhamnosus* (previously *Lactobacillus rhamnosus*) (Zheng et al., 2020), using protein extracted from *Ns*DSC and different biodegradable polysaccharides (inulin, alginate, or glucomannan) applying the layer-by-layer approach, and, in addition, to also determine the coatings’ effect on the viability of probiotic cells under gastrointestinal conditions and different heating temperatures.

## Materials and Methods

### Materials and Equipment

*L. rhamnosus* powder was purchased from Dicofarm (Italy). *Nigella sativa* defatted cake was purchased from Al-Hethnawi Company for oil extraction (Jenin, Palestine). Polysaccharides inulin and glucomannan were purchased from NOW Foods, United States, while alginate was from SIGMA, United Kingdom. The enzyme Activa^®^WM *Streptoverticillium* transglutaminase (TGase) was supplied by the Ajinomoto Co. (Tokyo, Japan). MRS agar, MRS broth, and 2.5-L anaerobic jars were from the OXOID company (Basingstoke, United Kingdom). Bile salts and bromelain enzyme from pineapple stems were brought from SIGMA (Dorset, United Kingdom). Peptone water was from DIFCO. The pieces of equipment were Memmert CO_2_ incubator, refrigerated centrifuge (ALC PK120R), Memmert shaking water bath, thermo mix (ONILAB HM100-Pro), drying oven (Raypa), microcentrifuge (Denever Instrument), pH meter 3310 (JENWAY), and autoclave (Hydra). Citrus peel low-methylated (7%) PEC (Aglupectin) was purchased from Silva Extracts s.r.l. (Gorle, BG, Italy). Vegetable glycerol (USP grade) was purchased from Heartlandvapes LLC., OK, United States. All other chemicals and solvents were of analytical grade.

### Bacterial Strain and Growth Conditions

Lyophilized *L. rhamnosus* was reconstituted in 10 ml of de Man, Rogosa, and Sharpe (MRS) broth, incubated anaerobically at 37°C for 48 h, then the process was repeated twice to assure purity. A 450-ml MRS broth was inoculated with 50 ml of the previous broth. The cultures were incubated as previously mentioned.

Bacterial cells were harvested by centrifugation (ALC PK120R) at 3,800 × *g* for 20 min at 4°C. The cell pellets were washed twice with sterile peptone water then twice with 0.15 M NaCl. The cell count of *L. rhamnosus* was performed using a standard plate count method using MRS agar under anaerobic conditions at 37°C for 48 h.

### Protein Extraction From *Nigella sativa* Defatted Cake

Protein concentrate was extracted from *Nigella sativa* defatted cake by acid–base extraction method as described by Sabbah et al. (2020) with slight modifications. Dried *Ns*DSC was ground and dispersed in distilled water (DW) (1:20, w/v), pH adjusted to 12.0 with 1 M NaOH, and stirred at room temperature for 2 h at constant speed. The suspension was centrifuged (4,300 × *g*, 20 min), and the supernatant was collected; then pH was adjusted to 6.0 with 1 M HCl to form a precipitate. The precipitate was collected by centrifugation (3,800 × *g*, 20 min) and uniformly distributed on an aluminum foil sheet for drying at 30°C and 20% relative humidity. The obtained protein concentrate (PC) was ground to fine particles using household electric grinder.

To enhance the solubility and purity of the protein, the PC was treated with bromelain (Sigma, United Kingdom) as follows: 20 g of PC was suspended in 100 ml of DW, and pH was adjusted to 7.0; then 2.5 g of bromelain was added. The suspension was placed in a shaking water bath at 37°C for 4 h, then was boiled for 2 min to stop the enzymatic reaction. The precipitate was separated by centrifugation (4,000 × *g*, 20 min) and discarded; the supernatant was freeze dried. The powder that was obtained was named black seed protein (BSP).

### Determination of Total Protein Content Using Bicinchoninic Acid Protein Assay

To quantify the total protein content in the protein concentrate (PC), the BCA protein assay protocol described by He (2011) was followed. Initially, a working reagent solution (WRS) was prepared by mixing 50 parts of BCA (reagent A) with 1 part of BCA (reagent B) (50:1, A:B). Then 100 μl of PC was dissolved in 2.0 ml of WRS in order to get the sample to a WRS ratio of 1:20. An Eppendorf tube containing the mixture was covered with aluminum foil and incubated at 37°C for 30 min. After that, the tubes were kept at room temperature for 10 min before measurement. Absorbance at 562 nm was measured and compared with BSA standard curve to calculate the protein content and percentage.

### Preparation of Coating Solutions

Briefly, 2 g of BSP was dissolved in 100 ml of distilled water (DW) and stirred overnight for sufficient hydration. Polysaccharide solutions of inulin, alginate, and glucomannan were prepared by dissolving 2 mg/ml, then sterilizing by microfiltration. pH was adjusted to 6.5 by adding 1 M HCl.

### Layer-by-Layer Synthesis

The layer-by-layer encapsulation of *L. rhamnosus* was conducted according to a previous study (Wang et al., 2019) with some modifications. A cell-to-polymer solution ratio of 1:8 was obtained by adding 5 ml of concentrated cells into 40 ml of BSP. To induce gelation, TGase (8 U/g of protein), which is used to strengthen the BSP layer by catalyzing the formation of isopeptide bonds between glutamine and lysine, was added into the mixture (stirring for 30 min). The protein microcapsules were collected by centrifugation (3,800 × *g*, 15 min). Then they were washed twice with peptone water and then twice with NaCl (0.15 M), and recollected by centrifugation (3,800 × *g*, 15 min) after each wash.

Fresh protein microcapsules (2 g) were added into 20 ml of alginate solution, mixed, and stirred at constant speed for 30 min in a prewarmed 40°C water bath. Then microcapsules coated by alginate were collected by centrifugation (3,800 × *g*, 15 min) and washed twice with sterile peptone water, and then with NaCl solution (0.15 M) twice.

**FIGURE 1:**
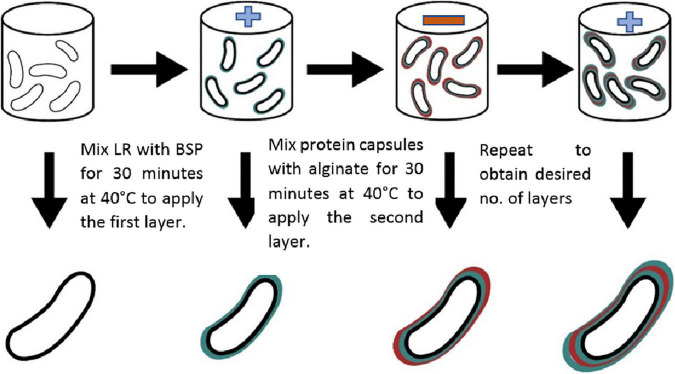
Layer-by-layer procedure to coat *L. rhamnosus* cells. Plain cells were mixed with BSP to apply first positively charged layer, a polysaccharide layer (alginate, glucomannan, inulin) was applied as second negative layer followed by a third layer of B SP.

As shown in Figure 1, the obtained microcapsules were coated by BSP to apply the third layer following the same steps. The same procedures were applied for BSP and replacing alginate with either inulin or glucomannan.

### Microscopic Examination of Layer-by-Layer-Coated Cells

Bacterial cells before coating and after LbL coating were examined under a light microscope (×1,000). Gram staining was applied, and any changes in the morphology of the cells were observed.

### Response of Layer-by-Layer-Coated Cells to Simulated Gastric Fluid and Simulated Intestinal Fluids

Survivability of plain and coated *L. rhamnosus* was evaluated after sequential exposure to SGF for 2 and 3 h to bile salt solutions (4%, w/v). One liter of stock, simulating gastric fluid solution, was prepared, using a previously reported method (Wang et al., 2019) with a few modifications. The solution contained 16.4 ml of hydrochloric acid (10%, v/v), sodium chloride (2 g), bromelain (10 g), and 983.6 ml of deionized water. The pH was adjusted to 2.8 using 1 M HCl and/or NaOH.

Bile salts (4 g) were added into 100 ml of deionized water, and pH was adjusted to 6.8, using 1 M NaOH and/or HCl, to form bile salt solutions (4%, w/v).

For digestion in SGF, the plain *L. rhamnosus* or LbL-coated cells were mixed with SGF and placed in a shaking water bath at 37°C. Samples were obtained every 30 min up to 120 min. Cells were pelleted *via* centrifugation (3,800 × *g*, 20 min), resuspended, and washed twice in peptone water. The suspension was serially diluted to an appropriate concentration, then the plain *L. rhamnosus* or coated cells were transferred to MRS agar plates and incubated anaerobically for 48 h at 37°C.

Sequentially, plain *L. rhamnosus* and coated cells (obtained after gastric simulation) were mixed with the 4% bile salt solution and placed in a water bath at 37°C. Similarly, samples were taken at 30, 60, 120, and 180 min. The cells were collected *via* centrifugation (4,000 × *g*, 20 min) and washed twice in peptone water as described previously.

The overall survival rate of *L. rhamnosus* was calculated after exposure to SGF and SIF by the equation of Li et al. (2016):

Survival (%) = log N*^t^*/N*^o^* × 100

where N*^t^* is the cell count at the end of different incubation times, and N*^o^* is the viable cell number prior to exposure to SGF and SIF.

### Heat Stability

The effect of the layer-by-layer coating on the heat resistance of *L. rhamnosus* was measured. Pelleted cells (1 g), prepared as mentioned above, were reconstituted in prewarmed peptone water using plain *L. rhamnosus*, (BSP-INU/BSP) *L. rhamnosus*, (BSP-ALG/BSP) *L. rhamnosus*, and (BSP-GLU/BSP) *L. rhamnosus* placed in water baths at 37, 45, or 55°C for 30 min to represent processing and storage conditions. Samples were diluted, and 1 ml was spread over MRS agar. The viable count of each sample was calculated after 48 h of incubation aerobically at 37°C. The results were expressed as log of cfu/g of the original sample.

### Incorporation of Layer-by-Layer-Coated Cells in an Edible Film

The LbL-coated cells were prepared as previously mentioned except that the final cell solution was concentrated to reach > 3 × 10^14^ cfu/ml, and BSP solution was 4%. Then the first layer was prepared by mixing the cell solution with the BSP solution in a 1:8 ratio. Inulin was used as the second layer followed by the BSP layer. The final LbL-coated cells were harvested and incorporated in a film as described by Giosafatto et al. (2019). Briefly, pectin (PEC)-based film forming solutions (0.8% w/v) were prepared from a PEC stock solution (1% w/v of water), at pH 7.0. LbL-coated cells (1.0%) were added to the PEC under continuous stirring for 30 min; after that, 30% GLY (w/w PEC) was added to the film-forming solution. The film-forming solution was casted onto 8-cm-diameter polycarbonate Petri dishes and allowed to dry in an environmental chamber at 30°C and 50% relative humidity (RH) for 24 h. The dried film was peeled, intact from the casting surface and tested for thickness and mechanical properties. A film of PEC without BSP was used as negative control, while a film of PEC with BSP/no LbL-coated cells was used as positive control for comparison.

### Film Characteristics

To compare the three different films, film thickness was measured with a micrometer screw gauge (0–25 mm, 0.1 μm) at different positions for each film sample. The films’ mechanical characteristics, tensile strength (TS), elongation at the break (EB), and Young’s modulus (YM) were analyzed by using the Brookfield CT3 Texture Analyzer (model CT3 50K, Brookfield, Chandler, United States) as descripted by Yaseen et al. (2021).

### Statistical Analysis

The experiment, starting from the LbL synthesis to the simulation process, was carried out in duplicate. In addition, the sampling for bacterial culturing also was performed in duplicate. All results obtained were analyzed using SPSS Statistics 21. Repeated measures, then one-way ANOVA test, with a *p*-value = 0.05, have been conducted.

## Results

### Total Protein Content

The percentage of total protein content in the original protein concentrate after enzymatic hydrolysis of NsDSC was 73.4%.

### Microscopic Features

**FIGURE 2:**
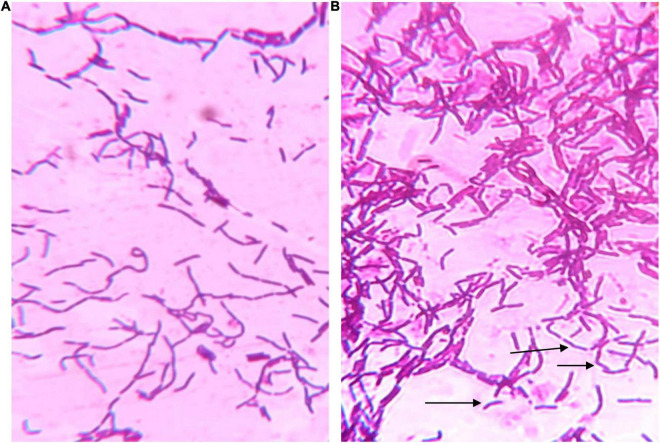
Microscopic photos of **(A)** uncoated cell and **(B)** LbL coated cells after gram staining. The coated cells were noticed to have clear areas around them as shown by arrows while uncoated cells were representing typical gram-stained bacteria.

Figure 2 shows that the LbL-coated cells were surrounded by clear areas. As the stain is positively charged, it can be repelled by the third layer of the coating (BSP), which is also positively charged. This has confirmed that the LbL coating was successfully applied.

### Survival of Plain and Coated *Lacticaseibacillus rhamnosus* Under Simulated Gastric Fluids and Bile Salts

**FIGURE 3:**
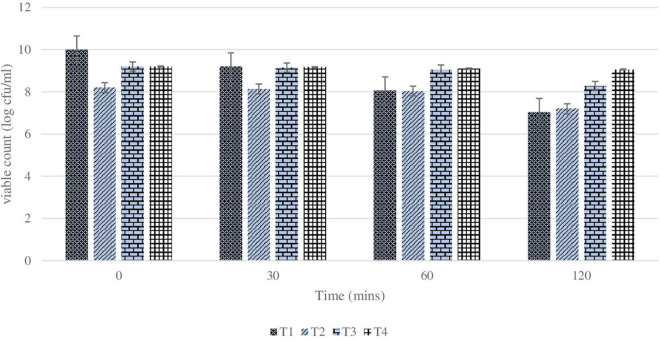
Changes in viability (cell count) of plain or coated *L. rhamnosus* cells after exposure to simulated gastric fluids for 30, 60, and 120 min. Tl: plain *L. rhamnosus*, T2: coated cells with BSP/Glucomannan, T3: coated cells with ESP/Alginate and T4:coated cells with BSP/Inulin.

**FIGURE 4:**
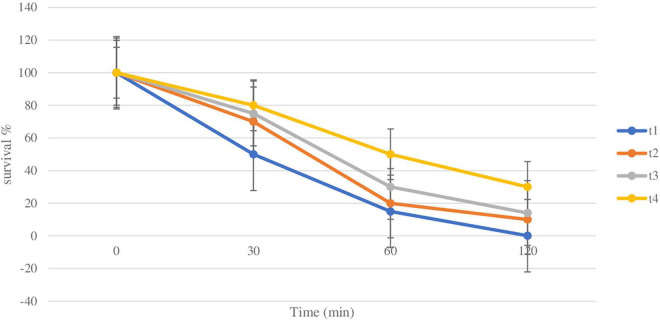
Survival rate of plain and coated *L. rhamnosus* under simulated gastric fluids after 30, 60, and 120 min. Tl: plain *L. rhamnosus*, T2: coated cells with BSP/Glucomannan, T3: coated cells with BSP/alginate and T4: Coated cells with BSP/inulin.

The main purpose of studying the survivability of plain and coated *L. rhamnosus* in SGF and SIF is to simulate the effect of low pH during transition through the GI tract. The effect of the coating of the *L. rhamnosus* on protection against SGF was tested by studying the differences in viability between plain and coated *L. rhamnosus* over varying time periods (30, 60, and 120 min). Since SGF treatment is carried out with fresh plain cells and BSP-based coated cells with inulin, alginate, and glucomannan, the initial cell counts of plain and coated cells were log 9.5 ± 0.5 and log 8.8 ± 0.6 cfu/ml, respectively, with survival rates of 50, 15, and 0.1%. As shown in Figure 3, the viability of plain *L. rhamnosus* was decreased to log 9.2, log 8.06, and log 7.04 cfu/ml, over 30, 60, and 120 min of exposure to SGF, respectively. While coating of *L. rhamnosus* with BSP and glucomannan showed better results, the viability reduced to log 8.14, log 8.04, and log 7.2 cfu/ml with survival rates of 70, 20, and 10%, respectively, over the same time periods. On the other hand, using alginate and inulin as coating materials with BSP confers a higher protection against low pH. In BSP/alginate-coated cells, viability reduced to log 9.15, log 9.06, and log 8.28 cfu/ml, and the survival rates were 75, 30, and 14% after 30, 60, and 120 min, respectively. Also, higher readings were obtained, log 9.16, log 9.1, and log 9.06 cfu/ml with% survival of 80, 50, and 30% over same time period, respectively, when *L. rhamnosus* cells were coated with inulin in the polysaccharide layer. Survival rates for plain and coated *L. rhamnosus* under SGF are presented in Figure 4.

**FIGURE 5:**
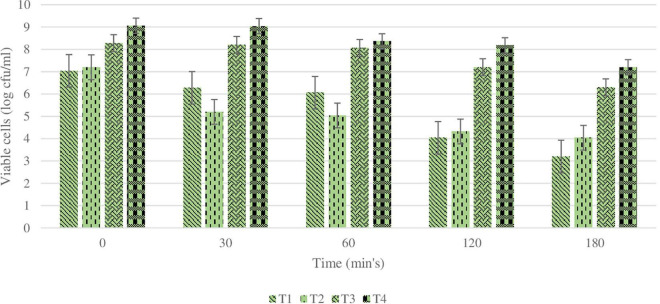
Changes in viability (cell count) of plain or coated *L. rhamnosus* cells after exposure to simulated intestinal fluids for 30, 60, 120, and 180 min. Tl : plain *L. rhamnosus*, T2: coated cells with BSP/Glucomannan, T3: coated cells with BSP/Alginate and T4:coated cells with BSP/Inulin.

**FIGURE 6:**
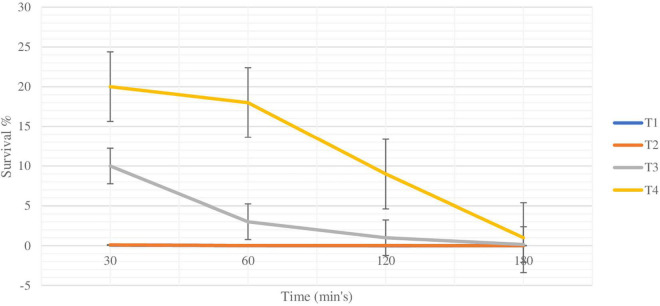
Survival rate of plain and coated *L. rhamnosus* under simulated intestinal fluids after 30, 60 120 and 180 min. Tl: plain *L. rhamnosus*, T2:coated cells with BSP/Glucomannan, T3: coated cells with ESP/alginate and T4: coated cells with BSP/inulin.

Since the major constituent of SIF is bile salts, the survivability of plain and coated *L. rhamnosus* in bile salts was assessed over varying time periods (30, 60, 120, and 180 min). As shown in Figure 5, in the presence of bile salts, the viability of the plain cells was reduced to log 6.28 l, log 6.06, log 4.04, and log 3.2 cfu/ml after 30, 60, 120, and 180 min respectively, with survival rates of 0.07%, 0.015%, 1 × 10^–4^%, and 5 × 10^–5^%. Over the same time periods, the viability of coated cells with BSP and glucomannan was reduced from log 7.2 to log 5.2, log 5.04, log 4.32, and log 4.04 cfu/ml, respectively, with survival rates of 0.1, 0.02, 0.016, and 0.002%. However, the resistance of cells coated with BSP/ALG or inulin showed higher results under the same conditions, while the BSP and ALG-coated *L. rhamnosus*’s viability reduced to log 8.2, log 8.06, log 7.2, and log 6.3 cfu/ml after 30, 60, 120, and 180 min, respectively, with survival rates of 10, 3, 1, and 0.15%. The viability of cells coated with BSP and inulin decreased from log 9.06 to log 9.04, log 8.36, log 8.18, and log 7.2 cfu/ml, and the survival rates were 20, 18, 9, and 1%, respectively, over the same time periods. Survival rates for plain and coated *L. rhamnosus* under SIF are presented in Figure 6.

### Heat Stability

**FIGURE 7:**
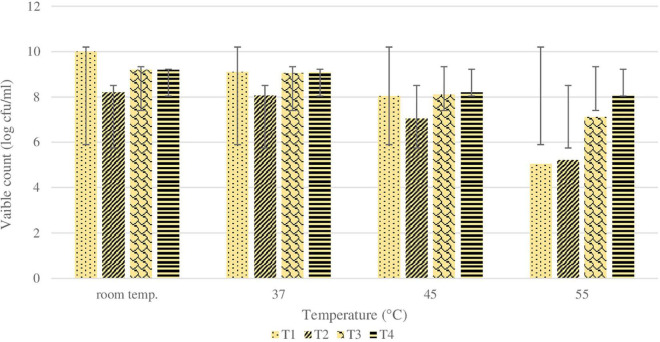
Changes in viability (cell count) of plain or coated *L. rhamnosus* cells after exposure to different temperature; room temperature, 37, 45 and 55°C for 30 min. Tl : plain *L. rhamnosus*, T2: coated cells with BSP/Glucomannan, T3: coated cells with BSP/Alginate and T4:coated cells with BSP/Inulin.

As mentioned previously, the initial count of 1 ml of plain *L. rhamnosus* was log 9.5 ± 0.5 cfu/gm. As shown in Figure 7, the cell count of coated cells with BSP and different types of polysaccharides (glucomannan, alginate, and inulin) were log 8.0, log 9.2, and log 9.2 cfu/g, respectively. After exposure of all samples to different heating temperatures of 37, 45, and 55°C for 30 min, the viability of both coated and plain *L. rhamnosus* was decreased differently.

**FIGURE 8:**
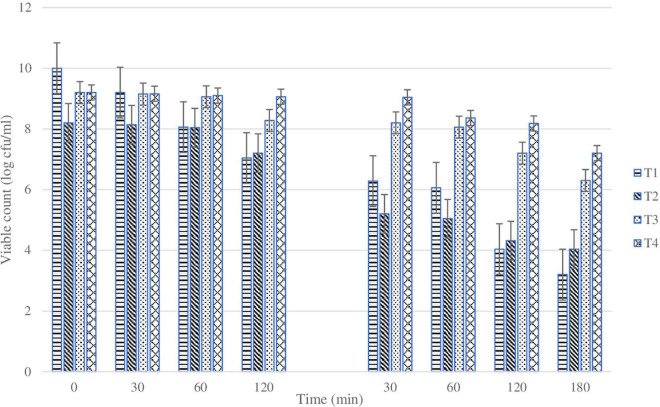
Changes in viability (cell count) of plain or coated *L. rhamnosus* cells after exposure to sequential gastric conditions for 30, 60, and 120 min. Tl: plain *L. rhamnosus*, T2: coated cells with BSP/Glucomannan, T3: coated cells with BSP/Alginate and T4:coated cells with BSP/Inulin.

Generally, LbL coating of cells showed higher survivals compared with plain cells. Particularly, survival (%) of plain *L. rhamnosus* was 25, 1, and 0.001% at 37, 45, and 55°C, respectively. Under the same temperatures, the cells coated with BSP and glucomannan showed 30, 2, and 0.1% survival at 37, 45, and 55°C, respectively. Moreover, the presence of alginate or inulin revealed a higher tolerance against heating temperatures. For BSP and alginate-coated *L. rhamnosus*, the survivability reduced to 30, 5, and 0.6%, while results for BSP and inulin-coated *L. rhamnosus* were 40, 10, and 3% at 37, 45, and 55°C, respectively. The statistical analysis results for sequential exposure to SGF and SIF are presented in Figure 8, which clearly shows that there was a huge reduction in the viability of plain *L. rhamnosus* when exposed sequentially to SGF and bile salts. The viability of plain cells was reduced almost by log 6 cfu/ml along the process, as previously described, while variations in viability reduction were shown depending on the polysaccharide incorporated. There were significant differences (*p* < 0.05) between treatments under sequential SGF and SIF compared with plain cells, in addition to significant differences (*p* < 0.05) observed between coating treatments.

Moreover, the significant differences obtained by statistical analysis of viable count of plain and coated cells when exposed to different heating temperatures are presented in Table 2. There were differences that existed between coating treatments and plain cells, in addition to differences between the coating treatments.

**TABLE 2:** Viable count of *L. rhamnosus* (mean log cfu/ml) when exposed to different heating temperatures for 30 min.

Parameter	Temperature	Plain	Glucomannan	Alginate	Inulin
Viable count under Heat treatment (log cfu/ml)	37°C	9.095^a^	8.06^b^	9.095^a^	9.085^a^
	45°C	8.04^c^	7.035^d^	8.1^b^	8.205^a^
	55°C	5.035^d^	5.3^c^	7.12^b^	8.06^a^

*Values in the same raw with different superscript are significantly different (p < 0.05).*

**FIGURE 9:**
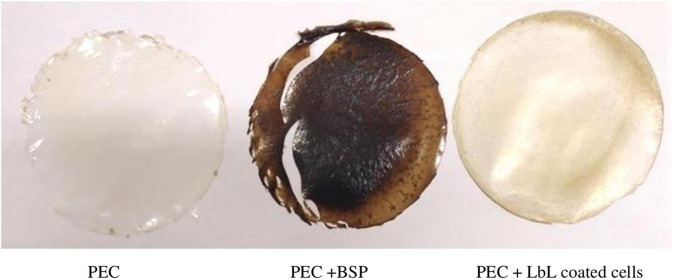
Images of pectin films (plain = PEC), with (BSP) or with LbL coated *L. rhamnosus.* cells were coated with BPS/inulin then used as part of pectin film. LbL containing film was superior over PEC+BSP in terms of handling and brittleness.

On the other hand, Figure 9 shows the three types of films prepared with or without LbL-coated cells. The results showed that the film containing BSP only was brittle, whereas film containing LbL had good handling properties. Moreover, the results reported in Table 3 indicate that the PEC film containing LbL showed higher thickness and YM values although not significant, while the TS (9.1 ± 0.4 and 13.6 ± 1.4 MPa, for PEC and PEC with LbL-coated cells, respectively) and EB (28.0 ± 6.8% and 37.1 ± 4.1% for PEC and PEC with LbL-coated cells, respectively) values were significantly higher compared with the control film obtained with PEC alone.

**TABLE 3:** Effect of incorporation of LbL microencapsulated *L. rhamnosus* on PEC film thickness and mechanical properties*.

Type of film	Thickness (μ m)	TS (MPa)	EB (%)	YM (MPa)
PEC	75.7 ± 2.1	9.1 ± 0.4	28.0 ± 6.8	36.8 ± 13.2
PEC with LbL coated cells	81.7 ± 4.5	13.6 ± 1.4*a*	37.1 ± 4.1*a*	37.2 ± 5.5

*The results are expressed as mean ± standard deviation. “a” significantly different values as compared to the PEC film obtained under the same experiment conditions.

## Discussion

The method used here showed high production of protein from black seed cake. Such high percentage is needed to make sure that the layer will be positively charged, as other impurities may interfere with this property.

Viability of commercial probiotics under the GI conditions was reported to be low (Millette et al., 2013). The effect of LbL coating on the viability of single-cell probiotics and its protective properties against harsh conditions are still under study and not hugely investigated. There were only a few studies regarding this method in the last 10 years. No single study, as far as we know, has investigated the use of the food industry byproducts as LbL coating materials for probiotics.

Generally, all studies previously published approved the positive effect of coating on the protection of probiotics in bacteria and yeast. Particularly, the LbL approach also enhanced the viability of probiotics under harsh conditions. The newest study, as far as we know, was made by Xiao et al. (2020) and published at the beginning of 2020. They investigated the effect of coating of *L. bulgaricus* and *L. paracasei* with whey protein isolate and xanthan gum using the LbL technique. The results revealed an increase in survivability and significant decrease in mortality under simulated GI fluids of coated cells compared with plain cells. In addition to higher tolerance to heating reductions have been recorded for coated cells at the same temperature. The same findings were supported temperatures, while 6 log reductions have been obtained when plain cells were exposed to 75°C, only 3 log previously by others (Priya et al., 2011; Cook et al., 2013; Thomas et al., 2014; Wang et al., 2019).

In LbL coating, the role of ionic interaction is essential to get better results. BSP is a positively charged polymer, which can easily form polyelectrolyte complexes with negatively charged polymers including alginate and inulin. Coating, in general, with proteins has a positive effect on the protection of probiotics under gastric conditions, pH 2 and 37°C (Picot and Lacroix, 2004; Gbassi et al., 2009; Doherty et al., 2011; Gerez et al., 2012; Maciel et al., 2014; Chen et al., 2017; De Araújo Etchepare et al., 2020; Maleki et al., 2020). Specifically, reduction of 2.8–3.9 logs of coating *Lactobacillus acidophilus* La-14, depending on the number of coating layers under gastric conditions, has been obtained, while a 7-log reduction was observed in the case of free cells (De Araújo Etchepare et al., 2020). These results are consistent with that of Gbassi et al. (2009) who found that coating of alginate beads with whey protein significantly improved the survivability of probiotics under acidic pH. Maleki et al. (2020) worked on mixing different encapsulating agents, whey protein isolate, inulin, and crystalline nanocellulose, in different concentrations to investigate the effect on protecting *Lactobacillus rhamnosus* ATCC 7469. They adduced that a higher WPI concentration mixture conferred a higher protection against simulated gastric conditions.

Alginate is the most widespread negative-charged polysaccharidein microencapsulation research and applications. The importance of alginate is linked to gelation, viscosity, and stabilizing properties, that alginate attributes to the product in which it is used.

On the other hand, the hydrophilicity of alginate may be partially disadvantageous; usage of high molecular-weighed alginate leads to undesirable properties related to viscosity in some products. The LbL technique has proven its ability to overcome this problem by using the minimum quantity of polysaccharides rather than use it as a core material.

Probiotic cells that are coated with only an alginate layer exhibit a higher releasing rate at acidic pH. This is explained by the higher porosity of alginate that has not been treated or coated with other coating material (Chávarri et al., 2010; De Araújo Etchepare et al., 2020). Dehkordi et al. (2020) reported that the survivability of *L. acidophilus* was 40% higher when the alginates are bead coated with whey protein isolate. In the current study, the alginate incorporation showed a better protection against harsh conditions and heating than the glucomannan did.

Indeed, incorporation of alginate as a coating material of *L. rhamnosus* showed a no significant reduction compared with plain *L. rhamnosus* after 30 min of SGF. However, a significant difference (*p* < 0.05) appeared after 60 min and continued to the end of the SIF process. However, it has a weaker effect compared with inulin, which was acting as alginate until 120 min of SGF; then the significant difference (*p* < 0.05) appeared with alginate-coated *L. rhamnosus* until the end of the GI simulation process.

Inulin is a negatively charged non-digestible polysaccharide, which is considered as a prebiotic, that selectively stimulates the growth and activity of probiotics in the colon. The addition of prebiotics significantly provided a better protection with only 3.1 and 2.9 log reduction for *L. acidophilus* 5 and *L. casei* 01, respectively, after incubation in simulated gastric juice at pH 1.55 (Krasaekoopt and Watcharapoka, 2014). In the same context, a significant improvement was reported in the viability of *L. casei* and *B. bifidum* when the capsules contained prebiotics, such as inulin (Zanjani et al., 2014) and *L. lactis* encapsulated with alginate and prebiotics (Persian gum), as reported by Nami et al. (2020).

According to Gandomi et al. (2016), incorporation of chitosan–alginate beads with inulin showed significantly (*p* < 0.05) an additional positive effect in the protection of *L. rhamnosus* against stringent conditions. Furthermore, the cooperation of inulin with WPI has provided a positive effect on the viability of Lactobacillus rhamnosus ATCC 7469 (Maleki et al., 2020). In the current research, incorporation of inulin as a coating layer beside BSP enhanced significantly the survivability of *L. rhamnosus* under acidic pH and bile salts as described earlier.

Glucomannan is a natural, water-soluble polysaccharide, which is considered an important component for the food industry, biopharmaceuticals, chemical industries, and other fields associated with human health. However, as shown in the results, the effectiveness of glucomannan on viability under low pH and bile salts was the lowest, owing to its non-ionic nature. These findings were partially in agreement with those of Nualkaekul et al. (2013), who observed the disability of glucomannan to protect alginate beads or improve cell survivability, while in the present study, it has affected positively the survivability but not as well as the other coating polysaccharides. The viability of glucomannan-coated *L. rhamnosus* was, surprisingly, lower than plain *L. rhamnosus* during the first 60 min under SIF. Then a significant difference (*p* < 0.05) was noted after 180 min of the simulation process.

When the plain and coated *L. rhamnosus* were exposed to different heating temperatures, the effect of temperature has significantly (*p* < 0.05) decreased viability as shown previously in Table 2. This may relate to the crucial differences in mechanism of protection of each polysaccharide, where glucomannan has a non-ionic nature leading to poor protection, alginate reduces the porosity of the surface, and inulin provides prebiotic activity, enhances the recovery of injured cells, and reduces the porosity.

The obtained mechanical values of PEC film results were in agreement with those of Jantrawut et al. (2017), who found that using 30% GLY with low methylated pectin resulted in a TS of about 5.0 MPa, EB of about 28.0%, and YM value of about 19.0 MPa. In our experiment, TS was increased from 9.1 ± 0.4 to 13.6 ± 1.4 MPa when LbL was incorporated to PEC at pH 7.0. This increase was partly due to the interaction of LbL with PEC polymers (Amor et al., 2021). Finally, production of functionally edible films containing LbL-coated probiotics, such as *L. rhamnosus*, was successfully achieved with promising properties for food or pharmaceutical applications.

## Conclusion and Future Perspectives

In summary, coating of probiotic strain *L. rhamnosus* was successfully performed using the layer-by-layer technique with BSP extracted from *Nigella sativa* defatted seed cakes and different types of polysaccharides (glucomannan, alginate, and inulin). In comparison with plain *L. rhamnosus*, coating materials have shown significant improvement against sequential exposure to SGF and also when exposed to high temperatures that may represent storing conditions.

A. These findings support that layer-by-layer is a promising technique to maintain high viable count of probiotics through hosts’ GI.
B. Indeed, the polysaccharides used as coating materials affect differently how the probiotics act under the GI conditions. While glucomannan showed the weakest protection, inulin confers the highest protection, significantly.
C. Incorporating LbL-coated cells in an edible film is a promising application that can be used in a variety of food products.

In future studies, the effect of LbL coating of probiotics on adhesion to mucosal lining of the colon and further effect on the gut microbiota are warranted. In addition, more investigations are needed on the coating of probiotic cells with the LbL approach using different coating materials, preferably materials extracted from industrial wastes to reduce the burden on the environment.

Moreover, application of coated cells by LbL technology in food processing, including highly consumed items, such as bread, meat products, fruits, and vegetable-based products, and special-need products like vegan milk, gluten-free, and lactose-free products, with different probiotics strains and coatings is highly recommended.

## Data Availability Statement

The original contributions presented in the study are included in the article/supplementary material, further inquiries can be directed to the corresponding author/s.

## Author Contributions

MSb conducted the experiment. MA and GM contributed to the concept design, designed the laboratory work, and approved the writing of the final manuscript. MS contributed to the experiment, discussion, and final writing. All authors contributed to the article and approved the submitted version.

## Conflict of Interest

The authors declare that the research was conducted in the absence of any commercial or financial relationships that could be construed as a potential conflict of interest.

## Publisher’s Note

All claims expressed in this article are solely those of the authors and do not necessarily represent those of their affiliated organizations, or those of the publisher, the editors and the reviewers. Any product that may be evaluated in this article, or claim that may be made by its manufacturer, is not guaranteed or endorsed by the publisher.

## Abbreviations

LbL: layer-by-layer
BSP: black seed protein
NsDSC: *Nigella sativa* defatted seed cakes
SGF: simulated gastric fluid
SIF: simulated intestinal fluid
IBD: inflammatory bowel diseases
TGase: mTrans-glutaminase
BCA: bicinchoninic acid
IN: inulin
ALG: alginate
GLU: glucomannan.
